# Electrochemically Obtained Polysulfonates Doped Poly(3,4-ethylenedioxythiophene) Films—Effects of the Dopant’s Chain Flexibility and Molecular Weight Studied by Electrochemical, Microgravimetric and XPS Methods

**DOI:** 10.3390/polym13152438

**Published:** 2021-07-24

**Authors:** Vladimir Lyutov, Varvara Kabanova, Oxana Gribkova, Alexander Nekrasov, Vessela Tsakova

**Affiliations:** 1 Institute of Physical Chemistry, Bulgarian Academy of Sciences, 1113 Sofia, Bulgaria; vlyutov@ipc.bas.bg; 2 A.N. Frumkin Institute of Physical Chemistry and Electrochemistry, Russian Academy of Sciences, 119071 Moscow, Russia; kabanovavar@gmail.com (V.K.); oxgribkova@gmail.com (O.G.); secp@elchem.ac.ru (A.N.)

**Keywords:** PEDOT, polysulfonates, polyelectrolytes, electrochemical doping, EQCM

## Abstract

Electrochemically synthesized poly(3,4,-ethylenedioxythiophene) (PEDOT) films obtained in the presence of eight different polysulfonate dopants are comparatively studied by means of electrochemical quartz crystal microbalance (EQCM) and X-ray Photoelectron Spectroscopy (XPS). Differences with respect to oxidation and doping levels (OL and DL), polymerization efficiency and redox behavior are revealed based on the interplay of three factors: the type of the dopant (acid or salt form), flexibility of the polysulfonate chains and molecular weight of the polysulfonate species. For the rigid- and semi-rigid-chain dopants, use of the salt form results in higher OL and DL values and substantial involvement of solvent molecules in the course of polymerization and redox transitions whereas in the presence of their acid form compact PEDOT films with minor ionic-solvent fluxes upon redox transitions are formed. In contrast, use of the salt form of the flexible chain polysulfonates results in PEDOT with lower OL and DL in comparison to the corresponding acid form. Significant effects are observed when comparing flexible chain dopants with different molecular weights. From a practical point of view the present investigations demonstrate the large scope of possibilities to influence some basic properties of PEDOT (Ol and DL, intensity and type of the ionic and solvent fluxes upon redox transition) depending on the used polysulfonate dopants.

## 1. Introduction

Poly(3,4-ethylenedioxythiophene) (PEDOT) continues to be the subject of numerous investigations due to the large scope of potential applications ranging from solar cells, light-emitting diodes, sensors and bioelectronics to conducting textiles, supercapacitors, etc. Several reviews (see e.g., [1,2,3,4,5,6]), published in the last few years, outline different application-oriented aspects of this polymer conducting material. The application-oriented aspects correlate strongly with core properties such as structure, morphology, ionic and solvent transport, conducting and mechanical properties. One of the essential factors that may influence markedly the properties of the conducting polymer materials is the type of doping ions used in the course of the polymer synthesis. In the case of PEDOT the prevailing number of studies address the commercially available polystyrene sulfonate(PSS)-doped PEDOT. Less attention is yet devoted to the various possibilities to use the doping as a powerful tool for modifying the PEDOT properties. Studies on the ion incorporation of electrochemically synthesized PEDOT layers obtained in the presence of mixtures of polystyrenesulfonate (PSS), p-toluenesulfonate and a number of inorganic anions (perchlorate, bromide, chloride, nitrate, thiosulfate, etc.) have shown that PEDOT has a greater affinity for PSS than for p-toluenesulfonate or small anions [7,8,9,10]. It was suggested that the reason for PSS acting as the counterion was due to the polymeric nature of PSS and not the SO3- group solely [7,8]. This implies that polysulfonates with different chemical structure may strongly affect the properties of PEDOT. A comparative study of PEDOT layers obtained in the presence of dodecylsulfate (a dopant inducing hydrophobic properties), PSS (characterized with flexible polymer chain) and poly(2-acrylamido-2-methyl-1-propane-sulfonate) (PAMPS) (as a typical hydrogel forming agent) has revealed the marked differences in viscoelasticity of the electrochemically synthesized PEDOTs [11,12]. Furthermore, the role of several different types of polysulfonates for the properties of chemically and electrochemically synthesized PEDOT was extensively studied in a series of investigations [13,14,15,16]. Polysulfonates with different flexibility of the polymer chain-flexible-chain PSSA (acid), and PAMPSA (acid), semi-rigid-chain poly(4,4′-(2,2′-disulfonic acid)- diphenylene-iso-phthalamide) (i-PASA) and rigid-chain poly (4,4′-(2,2′-disulfonic acid)-diphenylene-tere-phthalamide) (t-PASA) and their sodium salts (PSSNa, PAMPSNa, i-PASNa and t-PASNa, respectively) were used for electrochemical polymerization of EDOT. UV-vis-NIR and Raman spectroscopic studies have revealed marked differences in the obtained PEDOT materials. Thus, it was found that bipolaronic fragments of PEDOT are hardly found at high anodic potentials in the films prepared in the presence of acid forms of the rigid-chain, amide-containing polyelectrolytes (i.e, i-PASA and t-PASA). In-situ Raman studies during EDOT electropolymerization in the presence of these two polysulfonic acids has shown domination and retarded delocalization of EDOT^•+^ radical cations, the latter determining the rate of polymerization. These results were commented in terms of the difference in the inter- and intramolecular interaction of the polysulfonates with main emphasis on the proton transfer from sulfonic to amide groups in the PASA resulting in the coexistence of opposite charges in these polysulfonate chains [15].

The aim of the present study is to obtain further information on the electrochemical polymerization and electrochemical activity (including ionic and solvent fluxes) of PEDOT synthesized in the presence of the above mentioned polysulfonic acids (and corresponding sodium salts) with different flexibility of the polymer chains and different (high and low) molecular weights. To achieve this goal, in-situ EQCM studies in the course of electrochemical polymerization and electrochemical redox cycling are combined with ex-situ XPS studies carried out with the same specimens. There are so far few combined EQCM and XPS investigations on PEDOT [17,18,19,20] used to identify the thickness of PEDOT [19], availability of additional species such as diamond [17], and Pb [18l]. The combination of these two methods provides the unique opportunity to obtain independent information for the doping and oxidation levels of the PEDOT materials. To our knowledge such a combined study on the redox behavior of PEDOT was published only by Savva at al [20] and concerns chemically synthesized PEDOT/PSS.

## 2. Materials and Methods

**Scheme 1:**
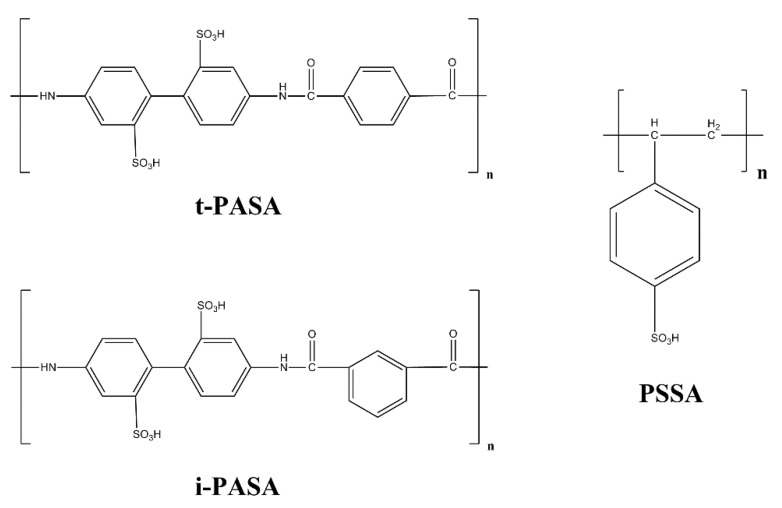
Polysulfonic acids used for the polymerization of EDOT.

The 3,4-ethylenedioxythiophene (EDOT), PSSNa (Mw = 1,000,000, 25% aqueous solution; Mw = 70,000, 30% aqueous solution), and LIClO4 were purchased from Sigma-Aldrich (now Merck Chemicals KGaA, Darmstadt, Germany) and were subsequently used for preparing all solutions for polymerization and characterization. The PASA polyacids were obtained in two steps—first by laboratory synthesis of i-PAS and t-PAS sodium salts [21] and as a second step converting them into the acids using an ion-exchange column. PSSNa was converted in H+ form by using a similar method. All polyacids or their corresponding sodium salts were purified via dialysis against distilled water (cellulose membrane ZelluTrans MWCO 8000-10000, Carl Roth, Karlsruhe, Germany). The corresponding molecular weights of the monomer units are: Mw = 184.14 g/mol for PSSA and Mw = 474.32 g/mol for t-PASA and i-PASA. The polyacids or their corresponding sodium salts were diluted with distilled water to obtain 0.02 M PSSA (or PSSNa), and 0.01 M i-PASA and 0.01 M t-PASA (or i-PASNa, and t-PASNa, correspondingly) aqueous solutions, that were left overnight on a magnetic stirrer. The concentration of i-PASA, t-PASA or their sodium salts was decreased two times in order to keep the ratio of EDOT monomer per sulfonic group constant in all solutions. EDOT was freshly distilled. Polymerization solutions were prepared by addition of 0.01 M EDOT in the corresponding polyelectrolyte solution. The process of the final solution blending took 1 h. The chemical structure of the polysulfonate dopants (in their acid form) used in the present study is shown in Scheme 1.

Poly(3,4-ethylenedioxythiophene) was electrochemically synthesized in the presence of the different polyacids or their sodium salts at a constant potential, E = 0.84 vs. Ag/AgCl by keeping the charge, Q_p_, for all PEDOT layers constant, Q_p_ = 25 mC. The redox behavior of the obtained PEDOT layers was investigated in 0.1 M aqueous LiClO_4_ solution by cyclic voltammetry at a scan rate of 50 mV/s.

A Metrohm-Autolab BV (Utrecht, the Netherlands) EQCM device coupled to Autolab 302 N potentiostat/galvanostat was used for simultaneous electrochemical and microgravimetric measurements. The working electrodes were gold-coated quartz resonators (6 MHz) with surface area of 0.361 cm^2^ and sensitivity of 4.43 µg/kHz. The polypropylene cell of the EQCM device was equipped with Ag/AgCl reference electrode and a gold wire counter electrode. Two parameters were collected in the course of the EQCM experiments—the frequency shift, Δf, of the quartz resonator and the so-called driving force, DF, (measured in V) necessary to initiate the oscillations. There is no straightforward interpretation of the last quantity but our experimental observations show that oscillation of electrode coatings with larger viscoelasticity require larger driving forces.

An AXIS Supra electronspectrometer (Kratos Analitycal Ltd., Manchester, UK) was used for X-ray photoelectron spectroscopy (XPS) measurements with base vacuum (~10^−7^ Pa) in the analysis chamber. An Al Kα achromatic radiation with photon energy of 1486.6 eV and a charge neutralization system was used for recording the spectra. The calibration of the energy scale was done by normalizing the C 1 s line of adsorbed adventitious hydrocarbons to 284.6 eV. The accuracy of determination of the binding energies (BE) was ±0.1 eV. The commercial data-processing software of Kratos Analytical Ltd. (Manchester, UK) was used to calculate the concentrations of the different chemical elements (in atomic %) by normalizing the areas of the photoelectron peaks to their relative sensitivity factors. The deconvolution of the high-resolution element spectra was implemented by the ESCApe. Software (Kratos Analytical Ltd., Manchester, UK).

## 3. Results and Discussion

### 3.1. Electrochemical Polymerization

**Figure 1:**
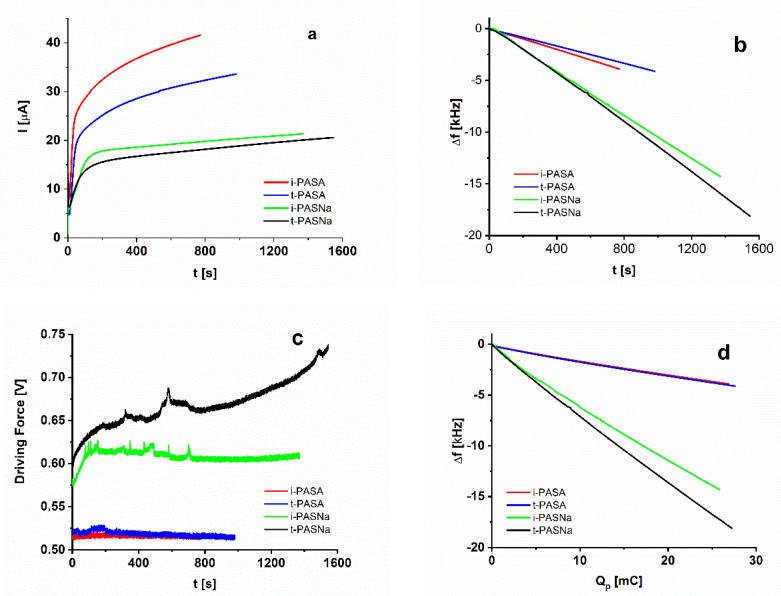
Current (**a**), frequency shift (**b**,**d**), and driving force (**c**) measurements in the course of potentiostatic polymerization in the presence of i-PASA (red) t-PASA (blue), i-PASNa (green) and t-PASNa (black).

Figure 1 shows results of the combined electrochemical and EQCM experiment obtained during PEDOT synthesis in the presence of the PAS-based dopants. It is apparent that the rate of polymerization (Figure 1a) is lower in the salt form of the polysulfonate solutions and this effect is accompanied by a significant negative frequency shift (Figure 1b), i.e., gain of mass due to doping polyanions and solvent molecules. On the other hand, in the acid PASA solutions the oxidation reaction is faster and a very low frequency shift is observed indicating to the formation of compact PEDOT/PASA layers. In fact, due to the low flexibility of both PASA and PEDOT chains, a structural matching effect could be expected for these polysulfonate/PEDOT couples. Furthermore, protons are expected to hop between adjacent sulfonic and amide sites [15,22], this effect resulting presumably in a limited charge compensating ability of PASA [15,16]. As a consequence, a lower level of doping (i.e., number of sulfonic groups per monomer units of PEDOT) should be expected. The data for the driving force (Figure 1c) reveal a smaller and constant value throughout the polymer growth for the two PASA dopants, whereas a significantly higher driving force is found in the PASNa solutions with sharp increase with time characteristic for the case of PEDOT/t-PASNa. The markedly different behavior of the t-PASNa dopant is also apparent in the Δf vs. Q_p_ plot (Figure 1d). Together with the sharp increase in the driving force observed at larger polymerization charges for the PEDOT/t-PASNa coating this should be an indication for larger viscoelasticity and a loss of proportionality between frequency shift and mass increase.

**Figure 2:**
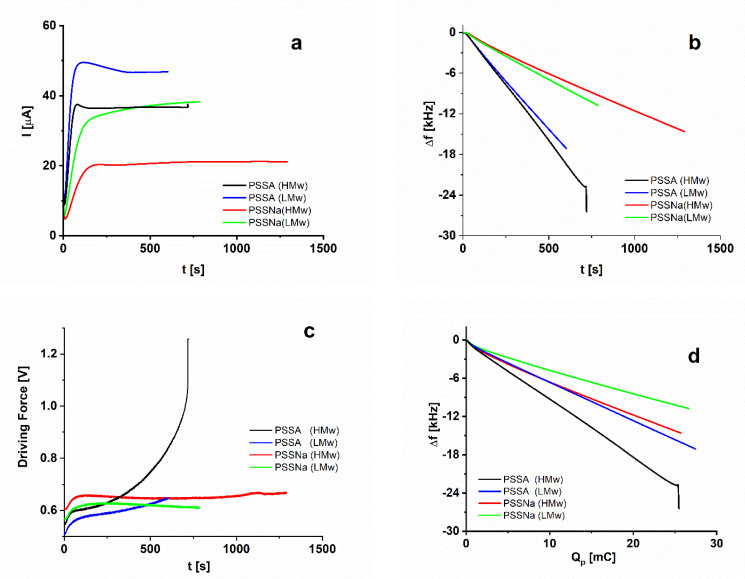
Current (**a**), frequency shift (**b**,**d**), and driving force (**c**) measurements in the course of potentiostatic polymerization in the presence of PSSA (HMw) (black), PSSA (LMw) (blue), PSSNa (HMw) (red) and PSSNa (LMw) (green).

A further set of experiments (Figure 2) was carried out in PSSA- and PSSNa-containing polymerization solutions by using corresponding high- and low-molecular-weight (HMw and LMw, respectively) dopants. According to Figure 2a the polymerization rate is again higher in the acid-dopant solutions in comparison to the salt ones. On the other hand, use of HMw dopants results in slower growth than of LMw ones, an effect that should be expected due to the larger solution viscosity in the case of HMw dopants. However, in contrast to the PASA-case, the fast growth in the PSSA-containing solutions is accompanied by a significant negative frequency shift especially in the HMw case (Figure 2b,d). At the final polymerization charge there is a four- to five-fold increase in the experimentally measured frequency shifts in the PSSA- compared to PASA-solutions (see Table 1). This implies a massive ingress of PSSA dopant and solvent species during polymerization that is due to the specific coil-like structure of the PSSA chains, in contrast to the expanded PASA chains. Furthermore, the HMw PSSA-doped PEDOT exhibits a high, steeply increasing values of DF (Figure 2c), a response that is characteristic for highly viscoelastic coatings. The growth of PEDOT in the PSSNa-containing solutions does not result in significant DF changes and the frequency shifts remain comparable to the PASNa-doped PEDOTs (Figure 2d and Table 1).

It is worth noting that for all specimens the frequency shifts depend linearly on the polymerization charge with small deviations from linearity as assessed by the regression coefficients of the linear fit (Table 1).

This marked difference in the behavior of PASA- and PSSA-doped PEDOTs implies the interference of a further factor, namely, the effect of pH and the type of charge-compensating cations on the conformation of flexible polyacid chains. The influence of the acidity on the PEDOT/PSS system was studied in several investigations [22,23,24,25,26] all showing a pH effect on the morphology, conductivity and UV-vis absorption spectra of the PEDOT/PSS materials. A pH-based reversible conductivity and morphology modulation of PEDOT:PSS composites was reported [24] with a suggested mechanism based on hydrogen bonds among the polymer composite system and the charge balance between PSS and PEDOT. Molecular dynamic simulations in the system PEDOT/PSSA [25] show a significant effect on the polymer composite morphology when varying the extent of PSSA protonation and thus the pH level between pH 0 and pH 5. Although a direct comparison of the characteristics of electrochemically deposited polymer thin films and the simulated composite PEDOT/PSS structures is not straightforward, apparently pH is a factor that influences to a great extent the conformation and charge compensation in these materials. The role of the different cations neutralizing the polyelectrolyte charge in the case of PSS- and t-PAS-doped PEDOTs was extensively studied [16]. It was shown that for flexible chain polysulfonates, such as PSS, the net negative charge along the chains increases in the order H ^+^ <Na ^+^ <Li^+^ which results in different degrees of chain coiling, the larger the net charge the more expended polyelectrolyte structure. Thus, the established differences observed in the presence of the PSSA and PSSNa should be related to the different conformation of these polysulfonates. In contrast, rigid-chain polyelectrolytes such as t-PAS preserve their conformation irrespective of the compensating cations [21]. Moreover, PASAs are amide-containing polyelectrolytes with different distance between sulfonic groups on the chain and accordingly different charge distribution along the chains. As a result, they have different mutual spatial organization of the macromolecules which significantly influences the structure and properties of PEDOT-PASs films.

### 3.2. XPS Studies

PEDOT/PSS obtained in chemical or electrochemical ways was investigated by XPS in several cases [7,8,27,28,29,30]. In these studies, the sulfur S(2p_1/2,3/2_) doublet spectra are usually considered and the observed peaks are assigned to sulfur atoms in different chemical environments, i.e., sulfur in PEDOT and sulfur in the sulfonic groups. The peak doublet corresponding to sulfur in the thiophene rings is found at the lowest binding energies with a tail on the high binding energy side due to sulfur atoms in the oxidized units of PEDOT. Three following doublet peaks are found to correspond to sulfur in three types of sulfonate groups: compensated by the positively charged PEDOT units, by Na^+^ or by H^+^ ions [27,28,29].

**Figure 3:**
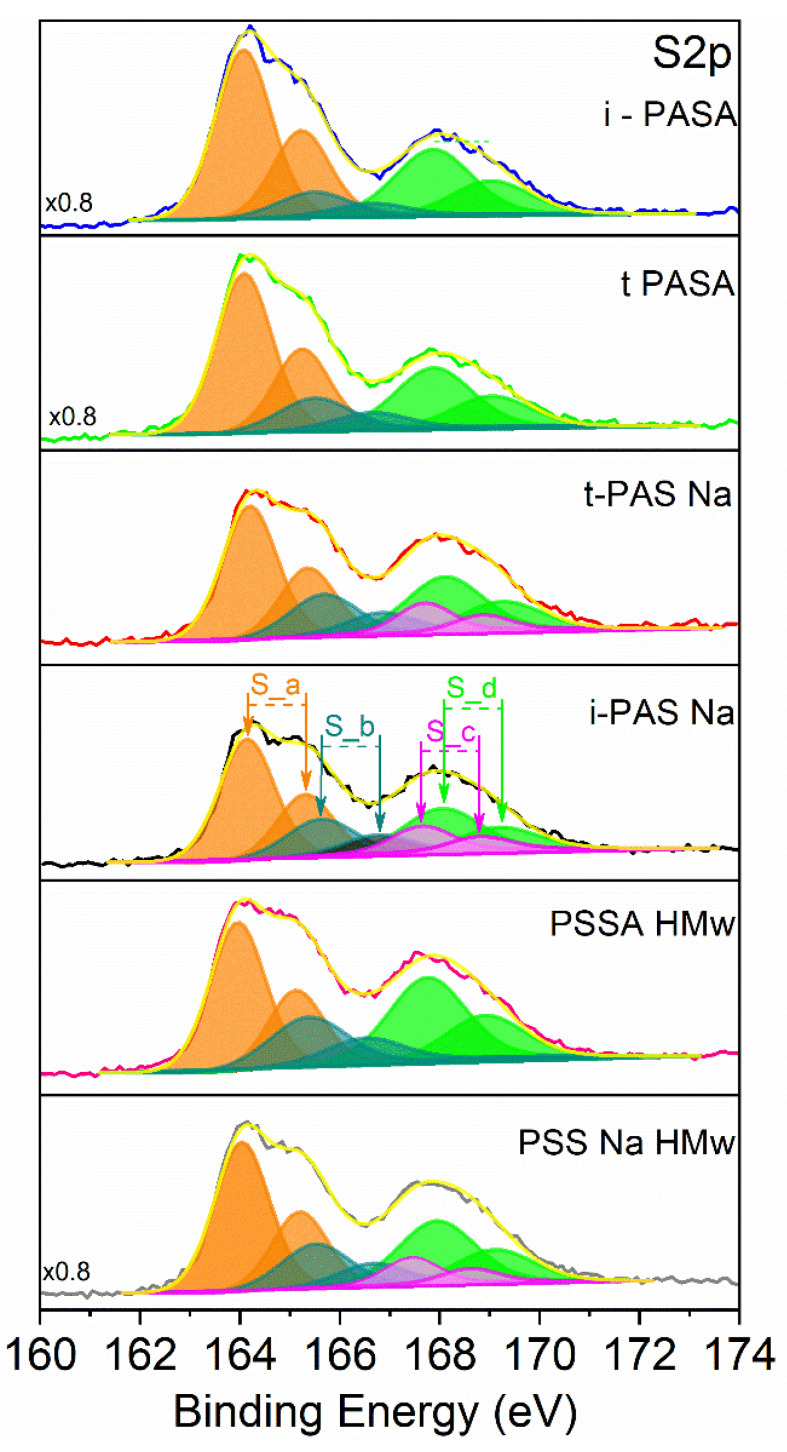
Deconvoluted S (2p) spectra of the different PEDOT materials.

XPS spectra were collected for six types of PEDOT specimens after electrochemical polymerization and voltametric cycling in neutral (LiClO_4_) solutions. Figure 3 shows the spectra with deconvolution of the sulfur doublet peaks. The energies of the separate peak doublets obtained by peak deconvolution are shown in Table 2 (the asymmetric tail used by Greczynski et al. [28] for fitting the high energy doublet peak has been neglected). As expected, the doublet in each peak couple is separated by 1.1 to 1.2 eV and sulfur of the sulfonate groups in Na^+^ and H^+^ environment by 0.4 eV [28]. The corresponding amounts of sulfur are presented in Table 3. Based on these values, data are obtained for the oxidation level, OL, (defined by the ratio of the amount of sulfur in the PEDOT-compensated sulfonate groups to the amount of sulfur in PEDOT) and the doping level, DL, (defined by the ratio of the amount of sulfur in all sulfonate groups to the amount of sulfur in PEDOT) (see Table 3).

The comparison of the OL and DL values of the two PAS-doped PEDOTs shows that for both quantities higher values are obtained in the salt PASNa solutions. Higher doping levels mean larger mass per unit polymerization charge, a result that is in line with the EQCM data showing larger frequency shifts in the salt solutions. Bearing in mind that PASA and PASNa contain one amide group per sulfonate group, for the PAS-doped PEDOTs values for DL can be calculated based also on the N-content (see last column of Table 3). For the PASA-doped samples there is a good correspondence between S- and N-based values of DL whereas for the PASNa-doped specimens the N-based DL values are somewhat larger. The calculations for the DL values account for all available types of sulfonic groups, i.e., compensated by PEDOT^+^, Na^+^ and H^+^, irrespective of the type of the used dopant (PASA or PASNa). Nevertheless, the doublet peaks of the sulfonic groups compensated by Na^+^ and H^+^ characteristic for the PEDOT/PASNa specimens are closely overlapping and this might impede the accurate evaluation of the peak area ratios.

In contrast to PAS-doped PEDOT, the PSS-doped PEDOTs show a different trend—both the OL and DL values are higher for the PSSA-doped specimen than for the PSSNa-doped one. This finding is again in line with the EQCM observations for these samples. XPS-based data for OL and DL were formerly obtained for electrochemically synthesized PSSA- and PSSNa- doped PEDOT films [29]. The values for both OL and DL were in this case lower amounting 0.35 and 0.25 for OL and to 0.68 and 0.98 for DL, for PSSA- and PSSNa-doped PEDOTs, respectively. It is worth mentioning that LMw PSSA and PSSNa were used in these experiments [29] and this should be the reason for the established lower oxidation and doping levels. Based on the observed correlation between doping level and frequency shift valid for all specimens investigated with both XPS and EQCM, a higher doping level should correspond to stronger frequency shift per unit polymerization charge. The data from the EQCM experiment with low- and high-molecular-weight PSSA and PSSNa (Figure 2d) show stronger frequency shifts for both types of dopants when using the HMw dopants.

The data for the doping level obtained from XPS studies provide the opportunity to estimate the possible lower PEDOT/polysulfonate layers weight limit. The theoretical mass per monomer unit of the dry polymers should be at least M = (M_EDOT_ − 2) + DL*M_polysulf_/n, where n is the number of sulfonate groups in the dopant monomer unit. On the other hand, the experimental mass per monomer unit, obtained from the EQCM data, M = mzF/Q_poly_, is expected to surpass the XPS-based value due to the indispensable presence of solvent molecules in the wet polymer coatings. The calculations (Table 4) carried out at Q_poly_ = 2 mC (in order to ensure the validity of the Sauerbrey equation) show that this is actually the case for all PASNa- and PSS-doped PEDOTs. As expected in most cases the experimental EQCM-based M values exceed the XPS-based ones but to a very different extent. A much larger effect of increased M (EQCM-based) values is observed for the PSS-doped specimens than for the PASNa-doped ones due to the larger ingress of solvent molecules accompanying the flexible chain PSS. Surprisingly, for the two PASA-doped specimens the XPS-obtained value of M is larger than the corresponding EQCM—based value. Such an effect should be attributed to a low polymerization efficiency which means that not all oxidized EDOT^•+^ species were incorporated in the growing polymer layer. In such a case the charge that is used for the calculation of EQCM-based values is overestimated and this results in smaller apparent values of the molar mass M. This finding supports the suggested role of the specific interactions in macromolecules of the rigid-chain polyelectrolyte in its acidic form resulting in partial inaccessibility of the sulfonic groups for charge compensation in PEDOT, thus destabilizing the formed EDOT^•+^ radical cations and preventing the growth of long PEDOT chains [15,16]. It could be also expected that under these conditions short oligomers become soluble in the polyelectrolyte solution.

### 3.3. Electrochemical Redox Activity—EQCM Studies

**Figure 4:**
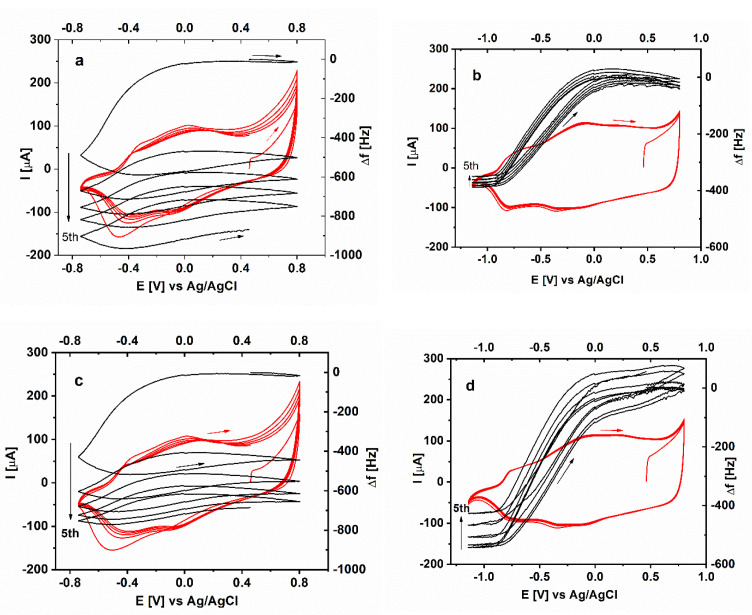
Simultaneous electrochemical (red) and microgravimetric (black) measurements in the course of five voltametric scans in aqueous solution of 0.1 M LiClO_4_ for PEDOT films electrodeposited in the presence of: (**a**) i-PASA, (**b**) i-PASNa, (**c**) t-PASA and (**d**) t-PASNa.

**Figure 5:**
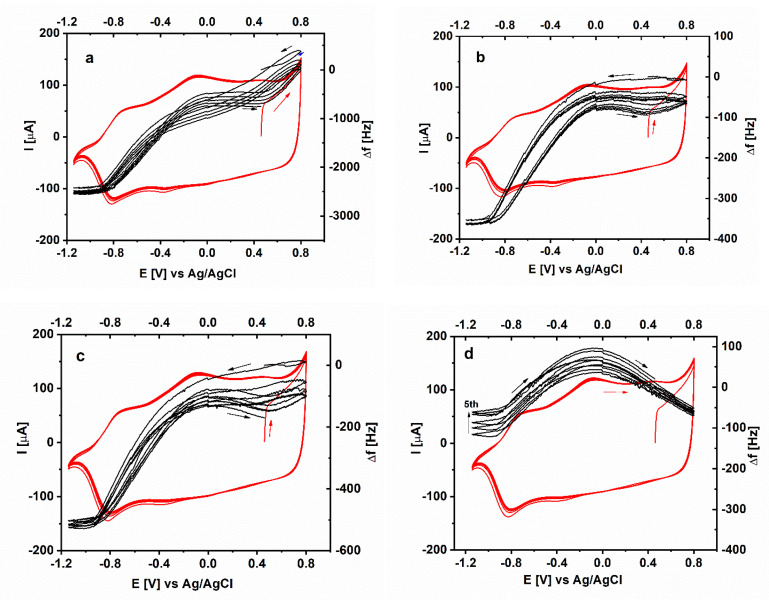
Simultaneous electrochemical (red) and microgravimetric (black) measurements in the course of five voltametric scans in aqueous solution of 0.5 M LiClO_4_ for PEDOT films electrodeposited in the presence of: (**a**) PSSA (HMw), (**b**) PSS Na (HMw), (**c**) PSSA (LMw) and (**d**) PSS Na (LMw).

The electrochemical redox behavior of all PEDOT specimens was studied in lithium perchlorate solution (pH = 6.2). Figs. 4 and 5 show combined (voltametric and EQCM) results obtained in the course of five consecutive voltametric scans. The voltametric measurements allow to estimate the charge exchanged during redox transitions, Q_ox_ = Q_red_. The data for Q_p_ and Q_ox_ (extracted from an individual voltametric curve) are often used to calculate the so called electrochemical redox number, y, by using the formula y = 2Q_redox_/(Q_p_ − Q_ox_). The data for y thus obtained are shown in Table 3. The comparison of the values for OL and y shows that the electrochemically obtained data are in all cases smaller than the XPS-based ones. There are two factors that may affect these results. The calculation for y presumes 100% efficiency of the polymerization process. In case of lower polymerization efficiency, the y values will be effectively underestimated. Furthermore, if the redox charge is experimentally underestimated which may easily occur due to the measurements in a dynamic voltametric regime this will result also in underestimated y values. The voltametric measurements under continuous cycling do not allow for a complete reduction of the polymer films and well-known effects such as presence of oxidized domains in an already reduced, non-conducting polymer matrix (i.e., trapped charges) or memory effects cannot be accounted for [31,32,33,34].

Let us further consider the evolution of the EQCM response within the five redox scans for the various PEDOT/polysulfonate specimens. The PASNa-doped PEDOTs (Figure 4b,d) show a slight positive frequency shift (loss of mass) in the course of cycling. It could be suggested that some of the initially available sodium ions become gradually exchanged by lithium ions. The molar masses of Na and Li differ by a factor of 3 but Li has a larger hydration shell than Na that should be also taken into account. Furthermore, accommodation of the polymer structure occurring at the repetitive redox transitions may be related to loss of solvent molecules. Such effects were already observed for PASA- and PSSA-doped polyaniline layers [35].

Surprisingly, instead of positive frequency shift (i.e., loss of mass), the two PASA-doped PEDOT layers show a significant negative frequency shift (i.e., gain of mass) especially in the first voltametric scan, a trend that is observed to a smaller extent also in the next cycles (Figure 4a,c). Bearing in mind the small frequency shift during polymerization as well as the constant driving force, established for these layers, it can be assumed that the Sauerbrey equation holds in this case. Thus, the gain of mass after the fifth voltammogram amounts to about 20% of the total initial mass of the as-synthesized PEDOT layers. In fact, for these layers exchange of protons for hydrated Li^+^ ions is expected to occur and this should result in higher weights in the oxidized state but not in the reduced state, which is obviously the case. So, it seems more realistic to assume that in the presence of hydrated Li^+^ the entire structure of the PASA-synthesized PEDOT specimens is forced to expand and ingress of additional solvent molecules can also contribute to the gain of mass.

**Figure 6:**
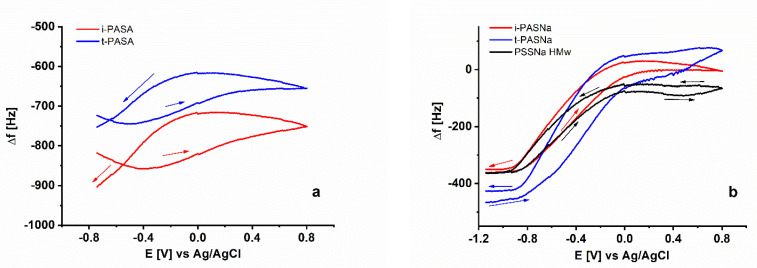
Frequency change as a function of potential in the course of electrochemical redox switching of PEDOT films obtained in the presence of (**a**) i-PASA (red) and t-PASA (blue), and (**b**) i-PASNa (red), t-PASNa (blue) and PSS Na (HMw) (black).

Looking at the individual voltametric cycle obtained for the four types of PAS-doped PEDOT layers (Figure 6) it is apparent that for the PEDOT/PASA specimens, despite of the large drift from scan to scan, only small amounts of mass become expelled/incorporated in the course of single oxidation/reduction cycle. The estimation of the mass change upon oxidation results in apparent molar mass of the expulsed species amounting to about 33 to 38 g/mol which is close to the molecular weight of two molecules of water. Basically, the PASNa-doped PEDOTs show the same trend of expelling of mass upon oxidation and ingress of mass upon reduction typical for cationic exchange. However, the apparent molar mass estimated upon oxidation amounts to 135 and 161 g/mol, respectively, and is much higher than for PASA-doped PEDOT. These values are larger than the molar mass of hydrated sodium ions (95 g/mol at hydration number of 4) or hydrated lithium ions (103 g/mol at hydration number of 6) and imply the ingress of several additional solvent molecules.

The experiment carried out with the PSS-doped PEDOTs (Figure 5) show that all PSS-doped specimens show a more stable behavior upon cycling in comparison to the PAS-doped ones. Due to the large frequency shift and the highly probable interference of viscoelastic effects the PEDOT/PSSA (HMw)-specimen will be excluded from further considerations although the characteristic response shown in Figure 5a is reproducible. Upon continuous cycling the PEDOT/PSSA(LMw) specimen shows almost no frequency drift in the reduced state whereas a weak shift to higher frequency is observed in the oxidized state.

It is worth noting that depending on the molecular weight the PSS Na-doped specimens show markedly different trends with respect to the frequency shifts upon redox transition. The PEDOT/PSS Na(HMw) specimen is characterized by a loss of mass upon oxidation with apparent molar mass of 119 g/mol which is somewhat less than for PEDOT/PASNa but basically in the same order of magnitude (Figure 6b). This result is close to the finding of Moderrasi et al. [26]. Based on molecular dynamic simulations of the system PEDOT/PSS it was suggested that upon redox cycling one sodium ion brings an average of four water molecules in the polymer structure. However, in contrast to all other polysulfonate-doped specimens, the LMw PEDOT/PSS Na shows first loss of mass up to a potential of about 0.0 V followed by mass regain with almost equal initial and final Δf values (Figure 5d). In general, this PEDOT material exhibits the smallest redox-driven difference in mass between initial and final state from all studied specimens. These effects should be related not only to simple charge compensation but also to changes in mutual spatial organization of the macromolecules and corresponding solvent fluxes that are much more difficult to be clearly resolved.

From a practical point of view the present investigation demonstrates the large scope of possibilities to influence some basic properties of PEDOT such as extent of oxidation and doping, intensity and type of the ionic and solvent fluxes upon redox transition depending on the used polysulfonate dopants. Thus, the properties of the PEDOT/polysulfonate system can be matched to specific requirements for their applications, e.g., avoiding the degradation of semiconducting devices due to the acidity of the PSSA-based PEDOTs [36] or achieving low or high mass exchange upon redox transition as required in different kinds of biomedical applications.

## 4. Conclusions

The present investigation on polysulfonate-doped PEDOTs showed consistent data obtained with the same specimen of a given type used for all types of measurements (electrochemical, EQCM and XPS). The comparative study of PEDOTs synthesized in the presence of eight different polysulfonate dopants allowed us to identify important differences with respect to several characteristics of PEDOT such as oxidation and doping levels, polymerization efficiency and redox behavior. The interplay of three factors, i.e., type of the dopant (acid- or salt), flexibility of the polysulfonate chains (rigid-chain in PASA or flexible-chain in PSS) and molecular weight of the polysulfonate species, were determined in combination with the properties of the conjugated polymer/polysulfonate couple.

For the rigid-chain and semi-rigid-chain PAS-based polysulfonates, the use of their salt forms resulted in higher OL and DL values and substantial involvement of solvent molecules in the course of polymerization and redox transitions. In contrast, the acid PASA-doped PEDOTs seemed to be very compact, highly rigid and with minor ionic-solvent fluxes upon redox transitions. The growth of these seemingly highly compressed structures occurred at low polymerization efficiency. The first redox cycles after polymerization were characterized by gain of mass presumably due to some expansion of the initial structure.

The flexible-chain PSS-based dopants showed a different behavior. Use of the salt solution results in PEDOT with lower OL and DL in comparison to the PSSA solution. Significant effects were observed when comparing PSS-based dopants with different molecular weight. Based on EQCM results the doping level of the PEDOT/PSS(HMw) exceeds the DL values of PEDOT/PSS(LMw). Furthermore, depending on the molecular weight the type of ionic transport seems to change from typically cationic (as expected for polyanionic-doped specimens) to a kind of mixed transport with equal initial and final weight observed only in the PEDOT/PSSNa(LMw) case.

In general, the present investigation provides new prospects to tune the PEDOT/polysulfonate system properties to specific target applications.

## Figures and Tables

**Table 1:** Final values of the frequency shift and parameters of the linear fit of the (−Δf) vs. Q_p_ dependences shown in Figure 1 and Figure 2 (plots d).

Dopant	−Δf (kHz)	d Δf/dQ_p_ (kHz/mC)	Intercept (kHz)	R^2^
i-PASA	3.900	−0.140	−0.130	0.9981
t-PASA	4.117	−0.143	−0.167	0.9977
PSSA (LMw)	17.087	−0.609	−0.480	0.9996
PSSA (HMw)	22.784	−0.904	−0.286	0.9991
i-PASNa	14.290	−0.547	−0.513	0.9975
t-PASNa	18.094	−0.660	−0.385	0.9994
PSSNa (LMw)	10.747	−0.388	−0.697	0.9996
PSSNa (HMw)	14.624	−0.546	−0.902	0.9964

**Table 2:** Binding energies in eV of the sulfur species in different chemical environments available in the PEDOT/polysulfonates.

S (PEDOT)	S (PEDOT^+^SO_3_^−^)	S (Na^+^SO_3_^−^)	S (H^+^SO_3_^−^)
164.1	165.4	167.7	168.1
165.2	166.5	168.9	169.2

**Table 3:** Data for the atomic percent ratio of the sulfur species found in the PEDOT/polysulfonate specimens and calculated values for the Oxidation and Doping levels (OL and DL) and the electrochemical redox number y.

Dopant	S (PEDOT) (% of S)	S (PEDOT^+^SO_3_^−^) (% of S)	S (Na^+^SO_3_^−^) (% of S)	S (H^+^SO_3_^−^) (% of S)	OL * (XPS)	y	DL ** S-Based	DL *** N-Based
i-PASA	58.6	10.8	-	30.6	0.18	0.16	0.71	0.64
t-PASA	56.4	14.5	-	29.1	0.26	0.17	0.77	0.77
PSSA (HMw)	45.0	20.2	-	34.8	0.45	0.27	1.22	-
i-PASNa	47.8	17.6	11.2	23.4	0.37	0.25	1.09	1.25
t-PASNa	46.3	17.5	10.1	26.1	0.38	0.23	1.16	1.40
PSSNa (HMw)	47.2	16.8	8.5	27.5	0.36	0.23	1.12	

* Oxidation level, OL = S (PEDOT^+^SO_3_^−^)/S(PEDOT). ** Doping level (S-based), DL = (S(PEDOT^+^SO_3_^-^) + S(Na^+^SO_3_^−^) + S(H^+^SO_3_^−^))/S(PEDOT). *** Doping level (N-based), DL = N/S(PEDOT).

**Table 4:** Comparison of data obtained by EQCM and XPS for the apparent molar mass corresponding to polymerization.

Dopant	M (XPS-Based) * (g/mol)	M (EQCM-Based) ** (g/mol)
i-PASA	307.64	214.05
t-PASA	323.41	219.31
PSSA (HMw)	365.09	1548.29
i-PASNa	411.03	1020.46
t-PASNa	427.84	1042.83
PSSNa (HMw)	370.64	1297.21

* M = (M_EDOT_ − 2) + DL*M_polysulf_/n. ** M = mzF/Q_poly_; calculated for Q_poly_ = 2 mC.

## Data Availability

Not applicable.
